# Notes of the Convention

**Published:** 1890-05

**Authors:** 


### NOTES OF THE CONVENTION.

The repeal of the per capita tax heretofore exacted of local societies was eminently just and timely. This the JOURNAL has urged and insisted upon for several years. We have pointed out the injustice of it, and showed how it operated to keep delegates away. It has been a thorn in the flesh. We know and have before cited instances where county societies have met and formally decided not to send representatives, because of the tax. It was a farce any way; for in the majority of cases, no tax was paid; and the few societies that did pay, paid fifty cents per capita, instead of $1 as stipulated in the by-laws.

Notwithstanding the JOURNAL has called attention to the omission, the nominating committee again failed to elect secretaries of the Sections; but left them to be appointed by the chairman, as before. The by-laws distinctly say the secretaries of all the Sections shall be elected like other officers. Up to date only one chairman has appointed his secretary and sent his name to the general secretary; and we presume it will be neglected as usual; so that the Transactions will be published with the secretaries all blank, as for the last two or three years.

A very sensible resolution was adopted urging chairmen of Sections to the performance of the duties expected of, and incumbent upon them, and requiring them to notify the secretary of their acceptance of the office. Failure to do so, forfeits the honor, and the president is called upon to make other appointments. To date Dr. Carhart is the only chairman who has complied with this requirement.

To be elected chairman of a Section, is next to being elected President. The chairman presides over the whole convention when his Section is called; it is resolved into a "Committee of the Whole." It is an honor; and the way in which some gentlemen have shirked every duty entrusted to them is simply shameful. We look for better things.

Another step forward was taken when the badges were placed in the hands of the treasurer and given out only on payment of dues. As a result of the first trial—there is more money on hand than ever before, at the close of a meeting; and after all expenses have been paid, there is enough to pay for a handsome Transactions, and leave five or six hundred dollars as a nest egg; something never before possible. Would any Doctor take a badge, wear it, and exercise all the rights and privileges of a member without paying his dues? Wouldn't he, though! A very considerable number at this meeting got out of paying altogether; and yet, got badges somehow. A member who has paid no dues in four years sent his little son to the secretary for a badge. The secretary wrote a note referring him to the treasurer, in whose keeping the badges were. He didn't get it; at least, he paid no dues.

The president's address was pronounced a masterpiece of English composition; it was classical, and literally sparkled with "gems of purest ray serene," new and original thoughts, uttered in choicest language. It was a prose-poem; and the writer of this heard a distinguished member—a State senator, say that had he derived no other benefit from the trip to Fort Worth, he would consider hsmself amply repaid for time and money in having heard that address alone. We regret that the "iron hand of the law" prevents the publication of it in the JOURNAL before it has appeared in the Transactions.

A lady physician was present as delegate from ——— County Society. She came purposely to present a paper on a subject ot some needed reform; but when she saw five hundred fine looking "grave and reverend signors" in convention assembled, and took in the scope of its possibilities,—or in the vernacular—"sized them up," her courage failed her. Said she, "this is a great deal bigger thing than I had anticipated; it is too big for my little paper." Quite a mistake she made; the paper would have received most courteous attention, and for one, we are sorry that she let her modesty outweigh all other considerations.

